# What Are the Best Practices for Co-Creating Patient-Facing Educational Materials? A Scoping Review of the Literature

**DOI:** 10.3390/healthcare11192615

**Published:** 2023-09-23

**Authors:** Isabella R. McDonald, Elizabeth S. Blocker, Elizabeth A. Weyman, Neil Smith, Andrew A. Dwyer

**Affiliations:** 1 William F. Connell School of Nursing, Boston College, Chestnut Hill, MA 02467, USA; mcdonaiw@bc.edu (I.R.M.); blockere@bc.edu (E.S.B.); weymane@bc.edu (E.A.W.); 2 “I Am HH” Patient Organization, Dallas, TX 75238, USA; neilsmith38@hotmail.com; 3 Massachusetts General Hospital—Harvard Center for Reproductive Medicine, Boston, MA 02114, USA

**Keywords:** co-creation, hypogonadotropic hypogonadism, Kallmann syndrome, patient education, patient participation, value healthcare

## Abstract

Co-creating patient-facing educational materials (PEMs) can enhance person-centered care by responding to patient priorities and unmet needs. Little data exist on ‘best practices’ for co-creation. We followed the Arksey and O’Malley framework to conduct a systematic literature search of nine databases (MEDLINE, PubMed, EMBASE, CINAHL, PsycINFO, Web of Science, Cochrane Library, Joanna Briggs Institute, TRIP—April, 2022) to identify empirical studies published in English on PEM co-creation to distill ‘best practices’. Following an independent dual review of articles, data were collated into tables, and thematic analysis was employed to synthesize ‘best practices’ that were validated by a patient experienced in co-creating PEMs. Bias was not assessed, given the study heterogeneity. Of 6998 retrieved articles, 44 were included for data extraction/synthesis. Studies utilized heterogeneous methods spanning a range of health conditions/populations. Only 5/45 (11%) studies defined co-creation, 14 (32%) used a guiding framework, and 18 (41%) used validated evaluation tools. Six ‘best practices’ were identified: (1) begin with a review of the literature, (2) utilize a framework to inform the process, (3) involve clinical and patient experts from the beginning, (4) engage diverse perspectives, (5) ensure patients have the final decision, and (6) employ validated evaluation tools. This scoping review highlights the need for clear definitions and validated evaluation measures to guide and assess the co-creation process. Identified ‘best practices’ are relevant for use with diverse patient populations and health issues to enhance person-centered care.

## 1. Introduction

Co-creation is broadly conceptualized as the process of creation through interactions with others [1]. In the context of healthcare, co-creation aims to create value in new goods and/or services by enabling interactions and exchange between diverse stakeholders (e.g., patients/families and healthcare providers). As such, co-creation is a collaborative and democratic approach that recognizes both patients and professionals as equal partners in finding solutions and creating value. Co-creation has been used across a variety of healthcare settings and with diverse patient populations to develop person-centered approaches to care that are responsive to patient-identified needs [2].

A person-centered approach to care considers the whole individual and recognizes patients/families as full, active partners in the design and implementation of healthcare [2]. Key tenets of person-centered care include empathy, respect, engagement, relationship, communication, shared decision making, holistic focus, individualized focus, and coordinated care [3]. Person-centered care is widely acknowledged as an essential component of quality care that can improve a variety of health outcomes, including physical and social well-being, patient knowledge, and satisfaction with care [2].

Co-creation is an aspect of person-centered care representing a low-cost solution to help improve healthcare delivery, including educating and activating patients/families for self-management [4]. Despite the growing use of co-creation in healthcare, there is a paucity of data on co-creation ‘best practices’. The purpose of this scoping review was to address the primary question, “what is known about co-creating patient-facing educational materials (PEM) with patients and/or families?”. We aimed to synthesize findings from the existing literature to identify ‘best practices’ that could serve as a roadmap to improve the co-creation of PEMs.

## 2. Materials and Methods

We conducted a scoping review guided by the Arksey and O’Malley framework [5]. There is no registered protocol associated with this scoping review. The literature search and review was conducted using Covidence™ 2.0 systematic review software (Veritas Health Innovation, Melbourne, Australia—www.covidence.org, (accessed on 23 December 2022)), and we report study findings using the Preferred Reporting Items for Systematic Reviews and Meta-Analyses extension for the reporting of scoping reviews (PRISMA-ScR).

### 2.1. Identifying the Research Question

The scoping review process was guided by a single primary question: “what is known about co-creating patient-facing materials with patients and/or families?”

### 2.2. Identifying the Relevant Literature

With the support of a research Librarian, we conducted literature searches (19 April 2022) in 9 databases (MEDLINE, PubMed, EMBASE, CINAHL, PsycINFO, Web of Science, Cochrane Library, Joanna Briggs Institute, and TRIP). The structured search used the medical subject headings (MeSH) terms and keywords “co-creation” OR “co-production” OR “co-design” OR “co-construction” OR “co-innovation” OR “co-build” OR “codesign” OR “co-establish” OR “collaborative health” OR “patient-directed” OR “patient-centered” OR “patient focused” OR “community based participatory research” OR “patient participation” OR “patient partnership” OR “user-centered design” OR “patient partnerships” OR “patient supported” OR “patient engaged consultative” AND “learning health system” OR “learning healthcare system” OR “patient-facing materials” OR “education materials” OR “patient materials” OR “patient resources” OR “psychoeducational tool” OR “information sheet” OR “patient education materials” OR “patient education” AND “patients” OR “clients” OR “support groups” OR “family members” OR “caregivers” OR “spouses” OR “parents” OR “subjects” OR “participants” OR “patient collaborators”. No language restrictions were placed on retrieving published articles.

### 2.3. Selecting the Literature

Eligible studies were published in English, had no date restriction, and reported on the process of co-creating patient-facing educational materials (digital or print) involving at least one healthcare professional and at least one patient or family member. Eligible studies could involve any healthcare discipline, disease entity, or research methodology (i.e., quantitative, qualitative, or mixed methods). Studies that only included patients/families as a final validation step were excluded. Articles retrieved from the structured literature search were imported into Covidence™ for screening. After removal of duplicates, all titles and abstracts underwent independent dual review (IRM, ESB, EAW). Subsequently, the remaining articles underwent independent, dual, full-text review (IRM, ESB, EAW). Any discrepancies during the review process were resolved by group discussion (with AAD).

### 2.4. Charting the Data

Investigators (IRM, ESB, EAW) independently extracted data using a structured form, and findings were cross-checked by another investigator. The structured form developed for this scoping review captured the country the study was conducted in, the topic/health issue, framework/guidelines employed, whether co-creation was operationally defined, outcome measurements/tools, summary of key findings, and author-identified strengths/weaknesses. Risk of bias was not conducted due to the methodological variability of included studies.

### 2.5. Collating, Summarizing, and Reporting Results

Extracted data from included articles were organized in a master table (Table S1). Findings were reviewed and analyzed using an iterative process to identify thematic elements [6] reflecting aspects that contributed to the study success. Identified themes were collapsed into categorical groups by discussion to identify ‘best practices’.

### 2.6. Synthesis of Results

Thematic categories of respective strengths (and respective weaknesses) were quantified to identify ‘best practices. The ‘best practices’ were organized along a timeline for the co-creation process to depict the natural sequencing of co-creation best practices.

### 2.7. Patient and Public Involvement

The final ‘best practices’ were reviewed by a patient leader (NS) who had previously participated in two projects co-creating PEMs. Discussion with the patient leader elicited feedback to refine and validate ‘best practices’.

## 3. Results

**Figure 1:**
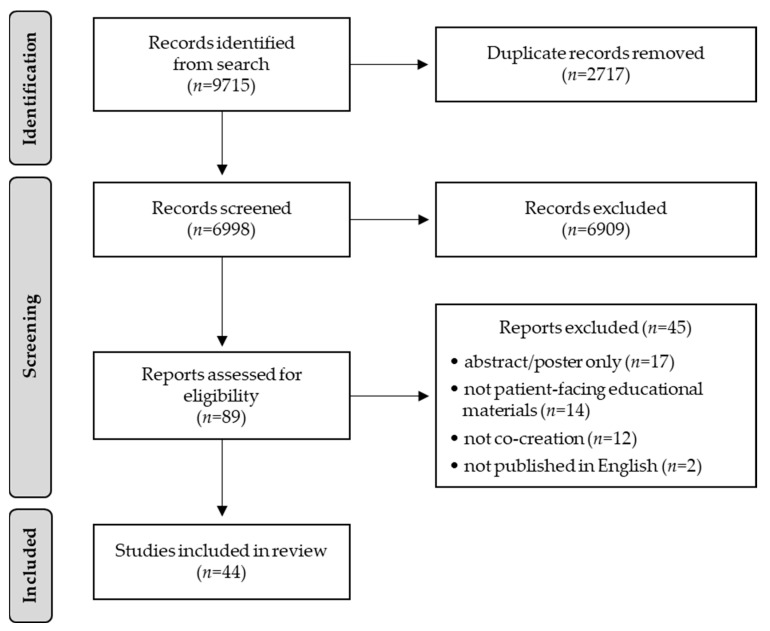
Scoping review PRISMA diagram.

The literature search identified 9715 articles, of which 2717 were duplicates, leaving 6998 articles for eligibility screening (Figure 1). A dual review of the title/abstract identified 6909 not meeting eligibility criteria, leaving 89 articles for full-text review. Dual full-text review removed 44 articles (abstract/poster only (*n* = 17), not patient-facing educational materials (*n* = 14), not co-creation (*n* = 12), not English (*n* = 2)), leaving 44 articles for data extraction. There was “substantial agreement” [7] in article selection (Cohen’s Kappa = 0.62). The full table of data extraction is provided in Supplemental Material (Table S1) [8,9,10,11,12,13,14,15,16,17,18,19,20,21,22,23,24,25,26,27,28,29,30,31,32,33,34,35,36,37,38,39,40,41,42,43,44,45,46,47,48,49,50,51].

### 3.1. Characteristics of Identified Studies on Co-Creation of PEMs

**Figure 2:**
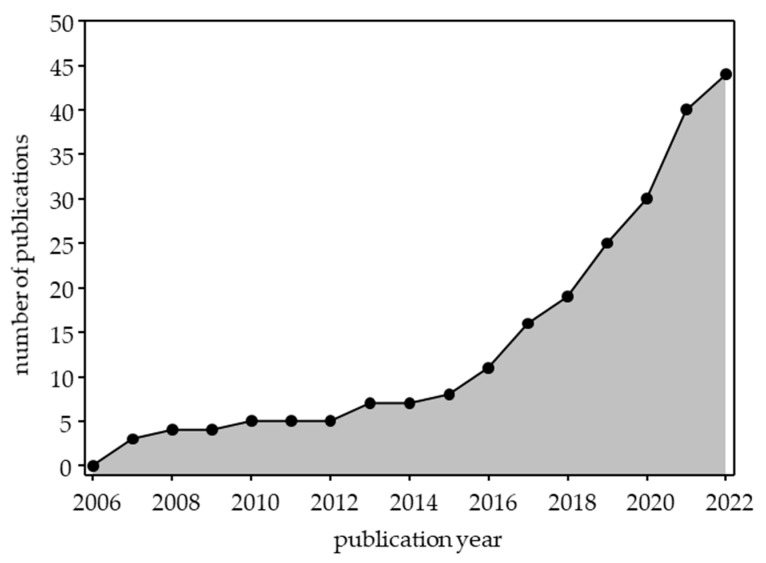
Cumulative publications on co-creation of PEMs by year (2006–2022).

Identified articles were published between 2007 and 2022, and 33/44 (75%) of papers were published since 2017, reflecting that co-creation is a relatively recent adoption in patient education (Figure 2) [9,10,11,12,13,14,15,16,17,18,20,21,23,24,25,26,27,28,31,34,36,37,38,40,41,43,44,45,46,47,48,49,50]. Three-quarters of papers were from anglophone countries—United States: *n* = 17 [8,9,17,19,20,22,25,27,31,32,33,34,36,42,47,49,50], Canada: *n* = 7 [10,13,18,28,43,44,51], Australia: *n* = 4 [14,21,46,48], Ireland: *n* = 2 [23,35], United Kingdom: *n* = 1 [39], with four publications from the Netherlands [11,12,15,38], two from Sweden [29,40], and one each from Denmark [45], France [26], Iran [41], Nigeria [30], Norway [37], Spain [16], and Switzerland [24]. In total, 42/44 (95%) articles were reported by groups from high-income countries (not high-income countries: [30,41]). Articles focused on diverse health conditions/patient populations, including specific health conditions, treatments/disease management, informed consent, and health promotion (Table S1). The most common category was cancer/oncology (*n* = 11) [10,11,14,18,20,31,38,40,41,47,50], followed by chronic health conditions (diabetes [19,23,27,29], cardiovascular disease [17,42,44,45] and chronic kidney disease/renal transplant [12,28,39,48], *n* = 4 each) and two publications each on asthma [22,32], inflammatory bowel disease [25,49], rare disease (congenital hypogonadotropic hypogonadism) [24,36], and transitional care (i.e., pediatric to adult-oriented care and hospital to home) [35,51]. Only 5/44 (11.3%) studies operationally defined co-creation [12,19,20,25,49].

### 3.2. Frameworks Employed to Co-Create PEMs

Most studies employed a framework or approach to guide the co-creation of PEM. However, nearly one-third (14/44, 31.8%) of studies [15,21,26,28,31,33,34,37,38,46,47,48,49,51] did not state a guiding framework. A range of terms were used to describe the approach to the co-creation process (Table S1). The most common approaches were ‘user-centered design/design thinking’ (10/30, 33.3%) [9,14,18,20,25,29,36,41,42,50] and ‘participatory approach’ (i.e., participatory action research, community-based participatory research, learning health system, patient-oriented research, or community engagement [10/30, 33.3%) [8,10,17,22,23,24,27,30,35,40]. One study used a combination of both a user-centered design and a participatory approach [19]. Other heterogeneous terminology was used to describe the approach to co-creation, including constructivist qualitative methodology [13,16,45], social cognitive theory [32], health action process approach [44], plan-do-study-act [39,43], patient empowerment model (interactive learning and action) [11], and an intervention mapping protocol [12]. Almost half (21/44, 47.7%) of studies involved patient partners from the very beginning of the co-creation process [14,17,19,20,21,24,26,28,29,31,34,36,37,38,40,41,46,47,48,50,51].

### 3.3. Approaches to Evaluate and Measure Effectiveness of Co-Created PEMs

In terms of measuring outcomes of co-creating PEMs, almost a quarter (10/44, 22.7%) of studies [9,11,20,23,25,26,30,39,40,43] did not employ an evaluation method and 4/44 (9.1%) studies [16,17,19,42] only reported on PEM development without reporting outcomes, as they were a component of an ongoing clinical trial. Of the 30 studies reporting outcome measures of co-created PEMs, qualitative methodology (i.e., user interviews, focus groups, open-ended survey responses, diaries, or “think aloud” exercises) was the most common approach, either alone or combined with another instrument (Table 1).

### 3.4. Author-Reported Strengths and Limitations of Co-Creation

Author-reported strengths and weaknesses were captured in the data extraction form (Table S1). Half of the studies (22/44, 50%) cited limited sample size, lack of diversity, and single-center recruitment as a limitation [8,9,10,11,12,13,16,18,20,23,25,27,29,32,34,35,36,41,45,46,47,50]. Additional limitations related to concerns regarding generalizability of findings (7/44, 15.9%) [8,11,15,23,27,30,32], recruitment challenges/possible recruitment bias (5/44, 11.4%) [14,18,24,31,40], and language concerns/English only (4/44, 9.1%) [14,24,28,48]. In relation to relative strengths or promoters of successful co-creation, nearly half of the studies (21/44, 47.7%) [14,17,19,20,21,24,26,28,29,31,34,36,37,38,40,41,46,47,48,50,51] noted including patients and healthcare professionals from the beginning as a promoter of successful co-creation of PEMs. Beginning the process with a literature review to understand the landscape of the issue was identified as a strength by 11/44 (25%) [8,19,20,24,26,28,31,33,37,46,51], and 10/44 (22.7%) [8,9,12,17,22,25,28,32,48,51] highlighted diversity in team members/patients as a strength supporting effective co-creation of PEMs. Three studies underscored that giving patients the “final say” in the co-creation process was important [23,24,35].

### 3.5. Synthesis of Findings to Identify ‘Best Practices’ for Co-Creating PEMs

**Figure 3:**
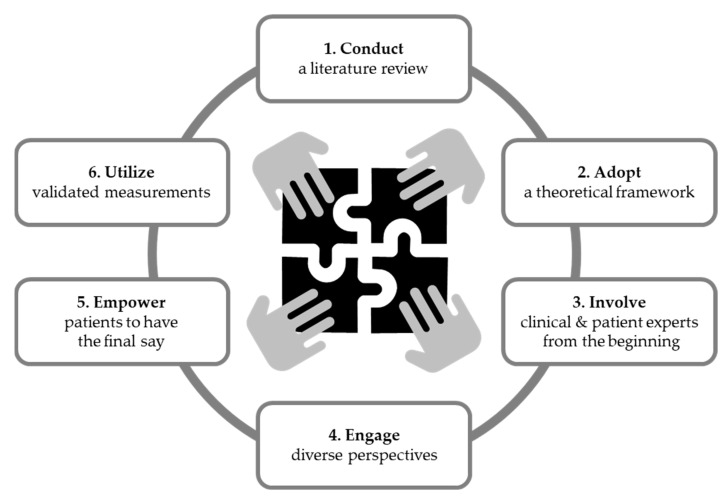
The scoping review of the literature identified six ‘best practices’ for co-creating PEMs.

Through iterative discussion, the research team synthesized the findings relating to the use of a guiding theoretical framework, relative strengths/weaknesses of the studies, and outcome measures to identify the salient themes guiding the co-creation process and supporting the development of high-quality PEMs (i.e., understandable, acceptable, and actionable). Six key themes were identified and mapped in a temporal manner to reflect the process from planning through creation to evaluation preceding implementation. The ‘best practices’ include (i) conduct a literature review, (ii) adopt a guiding theoretical framework, (iii) involve patients and healthcare professionals from the beginning, (iv) engage diverse perspectives in the process, (v) empower patients to have the final say, and (vi) utilize validated assessment tools (Figure 3). As a final validation step, we engaged a patient advocate (patient advocate from “I Am HH”) who had previously participated in two co-creation projects [24,36]. The patient leader provided critical feedback and noted that the identified ‘best practices’ are important for engaging patients, and support acceptability of co-created PEMs. Based on the feedback, the ‘best practices’ were deemed acceptable and reflective of practices that support patient-centeredness in the co-creation process.

## 4. Discussion

Our scoping review on co-creation of PEMs identified 44 articles from the systematic review of nine databases. More than half (24/44, 54%) of studies were from groups in North America (United States and Canada). Two-thirds (33/44, 75%) of papers were published since 2017, indicating that co-creation of PEMs is a relatively recent adoption in healthcare, with growing interest. Identified studies spanned a range of health concerns and patient populations, suggesting that co-creation is an adaptable, flexible approach with broad applicability. Co-created PEMs support effective patient education, underpinning patient activation and self-management. As such, using ‘best practices’ for co-creating PEMs holds relevance for an array of stakeholders, including patients/families, healthcare providers, health systems, and payors.

We identified six ‘best practices’ from our scoping review of co-creation of PEMs (Figure 3). First, conducting a literature review provides a deep understanding of the issue under examination, including current evidence and knowledge gaps. Eleven (25%) articles [8,19,20,24,26,28,31,33,37,46,51] reported that the co-creation was based on a literature review. All 11 articles used the literature review to gather available evidence and synthesize existing knowledge to inform PEM development. Notably, there are many types of reviews, and the literature review may be informal or take a more structured approach [52]. Understanding the current state of the science is a critical step for developing PEMs, as end-users may utilize information from PEMs to inform health decisions. Thus, providing accurate, up-to-date information based on the available evidence can support informed decision making. One goal of person-centered care is to engage patients in their healthcare decisions and to support high-quality decisions relating to an individual’s health and care [3]. High-quality decisions are both informed and aligned with one’s values and preferences. Accordingly, summarizing the best available evidence in language that is readily understandable by patients is one key component of supporting patients and families in making high-quality decisions.

Second, co-creating PEMs can be considered a complex intervention. Complex interventions are broadly considered as events that occur within systems (e.g., healthcare systems). Conceptualizing interventions in the context of systems facilitates understanding of the interactions between the interventions and the context in which it is implemented. The United Kingdom Medical Research Council recommends utilizing a guiding theoretical framework when planning and implementing a complex intervention [53]. Thus, utilizing a framework can inform and guide the co-creation process. We observed a range of guiding methodologies among the identified articles. The most frequently employed frameworks were user-centered design/design thinking and participatory approach (i.e., participatory action research, community-based participatory research, learning health system, patient-oriented research, or community engagement). Regardless of the framework employed in the respective articles, authors identified that utilizing a guiding theory or framework provided a dynamic perspective that considered many elements involved in co-creating PMs for the target audience within the specific context. Thus, utilizing a guiding framework can help dissect and address key elements that affect implementation and acceptability for co-created PEMs.

Third, involving both clinical and patient experts from the initial stages ensures that the process adheres to central tenets of patient-centered care (i.e., empathy, respect, engagement, relationship, communication, and shared decision making) [3]. Nearly half (21/44, 48%) of the identified articles included for synthesis involved patients from the beginning of the co-creation process [14,17,19,20,21,24,26,28,29,31,34,36,37,38,40,41,46,47,48,50,51]. The articles cited that including patient perspectives throughout the process helped inform priorities and helped center the final product on patient-identified needs and priorities. Presumably, the ongoing and iterative participation of patients contributes to producing PEMs that are understandable, acceptable, and responsive to patient priorities. Accordingly, involving patients from the beginning of the co-creation process may bolster the creation of person-centered PEMs.

The fourth key factor (engaging diverse perspectives) relates to involving an array of stakeholders with differing perspectives to help create value. Diversity reflects both the type of stakeholder (i.e., healthcare professionals and patients/families) as well as diversity in identity (i.e., race, ethnicity, age, education, etc.). Ten (23%) articles cited the inclusion of diverse perspectives as a strength of the co-creation process [8,9,12,17,22,25,28,32,48,51]. Authors noted that including diverse perspectives enhanced broader applicability and relevance to a wider stakeholder audience. Emphasizing diversity can support end-user acceptability and spur adoption to help overcome translation into practice [54].

Fifth, the notion of empowering patients to have the final say on PEMs additionally supports the patient-centeredness of co-creation. It merits noting that among the identified ‘best practices’, empowering patients to have the final say was only noted as an author-identified strength in 3 of the 44 identified articles. The modest number of supporting articles may be an artifact of our selection process, as 12 studies were excluded (Figure 1) for not meeting our definition of the co-creation process (i.e., involving patients from the beginning). The excluded studies used patients as a final validation step to approve the PEMs created by clinicians/investigators. Thus, excluded studies may provide indirect support for the importance of empowering patients to have the final say. Second, patients are experts in their condition and are in the ideal position to determine whether PEMs are understandable, acceptable, and actionable (i.e., high quality). Valuing patients/families as equal partners in finding solutions by creating space for their perspectives and opinions helps ensure that PEMs are grounded in patient priorities [55]. As noted by the patient advocate involved in this study, partnering with patients/families as equals helps support the relevance and acceptability of co-created PEMs. Involving the patient advocate in this scoping review provided key stakeholder support for including patient empowerment as a ‘best practice’.

Last, utilizing validated instruments represents an important step in improving the rigor of work on co-creating PEMs. Many studies used evaluation methods that were not validated or evidence-based. An important caveat is that validated instruments may not be available for some co-created products (i.e., disease-specific PEMs). Mixed-methods approaches employing validated instruments combined with qualitative methodology can provide in-depth information. However, it is worthwhile to note there are several validated, widely used instruments measuring constructs highly relevant to PEMs. Examples include readability algorithms (e.g., Flesch Reading Ease Formula, Flesch Kincaid Grade Level Formula, Gunning Fox Index, Coleman Liau Index, Simple Measure of Gobbledygook (SMOG), Automated Readability Index, and the Linsear Write Formula), health literacy/numeracy instruments (Short Assessment of Health Literacy (SAHL) [56], Rapid Estimate of Adult Health Literacy in Medicine (REALM) [57], rapid assessment of health literacy [58], Newest Vital Sign (NVS) [59], the System Usability Scale (SUS) [60], and the ‘gold standard’ Patient Education Materials Assessment Tool (PEMAT) from the U.S. Agency for Healthcare Quality and Research [61]. Employing validated tools helps limit bias in evaluation and can provide strong evidence that the materials are acceptable to patients.

This project involved a leader of a patient organization who had experience in co-creating PEMs [24,36] to validate the ‘best practices’ distilled from the literature. The patient advocate had three specific comments. First, in the context of rare disorders, PEMs may serve an important, dual role, as PEMs may also serve to inform clinicians—who may not be familiar with or have specific expertise in a particular rare disorder. Second, PEMs could be written by patients and then validated by HCPs. While no such PEMs were identified in our literature search, it seems plausible that patient organizations could create PEMs that could subsequently be validated by expert clinicians prior to dissemination. Last, the patient advocate noted that co-creation embraces patients as equal partners and thus empowers patients to share their lived experiences. As such, co-creation shifts the approach from a traditional hierarchical paradigm to one that respects and values patient perspectives. Co-creation draws on patient perceptions and experiences of their condition, care, and treatment. The development of patient-reported outcome measures (PROMs) typically utilizes qualitative data from patients to define key constructs and enhance content validity when creating PROMs [62]. As such, future directions could employ co-creation to develop PROMs.

Recent breakthroughs in natural language processing and large language models have helped generate powerful artificial intelligence and machine learning (AI/ML) tools (e.g., ChatGPT4). Such in silico tools have generated significant interest in healthcare. Recent provocative studies suggest that AI/ML chatbots can generate quality, empathetic responses to patient questions [63]. While there is excitement about these emerging technologies, it remains to be seen what role AI/ML may have in creating PEMs. Emerging AI/ML technologies are dependent on the data used to entrain the system, so it remains uncertain whether data sets available on the web could provide similar or equivalent results as involving patients in the co-creation process. Importantly, emotional empathy garnered from sharing one’s experience presents a unique challenge for AI/ML [64]. Sharing experiences is a central aspect of the co-creation process and can create a profound sense of mutual understanding and community.

### Strengths and Limitations

As co-creating PEMs is a relatively new phenomenon, we chose to conduct a systematic scoping review, as scoping studies are particularly relevant to areas with emerging evidence [65]. A relative strength of this work is the rigorous, systematic approach to conducting the scoping review that was guided by a well-established methodology. Further, incorporating multiple reviewer perspectives (including a patient with experience co-creating PEMs) increases our confidence in the reliability of the identified ‘best practices’. This study has several limitations. First, our scoping review only identified 44 published articles (2007–2022) reporting on the co-creation of PEMs. We distilled ‘best practices’ across the 44 studies, regardless of health condition or patient population. Having a larger body of published literature to draw from would have strengthened our process in identifying ‘best practices’. While we conducted a structured literature search of nine databases (using numerous terms/keywords), it is possible that not all studies were identified, and we did not conduct an extensive search of the grey literature. Indeed, while the TRIP database includes some grey literature, it should not be considered exhaustive. Additionally, findings may have an anglophone bias, as the majority of studies were published by groups in English-speaking countries. Further, two studies were excluded for not being published in English. As such, caution is merited in extrapolating findings to all cultural/linguistic contexts. Last, we considered co-creation to be a democratic process that involves patients/families as equal partners throughout the project. Accordingly, we excluded studies that involved patients/families at the end of the process as a validation step.

## 5. Conclusions

There is growing interest in using co-creation as part of forming more person-centered approaches to care. Our synthesis of the existing literature identified six ‘best practices’ for co-creating PEMs: (i) begin by conducting a literature review; (ii) adopt a guiding theoretical framework; (iii) involve patients and healthcare professionals from the beginning; (iv) engage diverse perspectives in the co-creation process; (v) empower patients to have the final say; and (vi) utilize validated assessment tools. Our findings indicate that co-creation is a flexible, broadly applicable approach to enhancing person-centered care that is relevant to wide-ranging health conditions and patient populations. Future directions include clarifying terminology used to describe co-creation, more widespread use of validated and evidence-based evaluation tools, and establishing a structured reporting guideline (i.e., EQUATOR Network) to facilitate comparability of co-creation projects in healthcare.

## Figures and Tables

**Table 1:** Outcome measures used alone or in combination to evaluate co-created PEMs.

Measure	Count	Reference(s)
Qualitative (constructivist)	15	[12,13,18,21,28,29,31,32,34,41,44,45,46,50,51]
Study-specific questionnaire	8	[8,10,12,14,22,35,46,47]
Web traffic/usage data	4	[34,35,38,49]
Validated disease-specific instrument ^1^	4	[15,27,34,50]
Validated readability algorithm ^2^	4	[24,33,36,48]
Validated health literacy instrument ^3^	3	[12,38,46]
Patient Education Materials Assessment Tool (PEMAT)	3	[24,36,48]
System Usability Scale (SUS)	2	[10,37]
Mobile Applications Rating Scale (MARS)	1	[48]
Patient Activation Measure (PAM)	1	[38]
Decisional Conflict Scale (DCS)	1	[10]
International Patient Decision Aids Standards (IPDAS)	1	[15]

^1^ Disease-specific instruments include: Glucose Monitoring Self-Efficacy Scale, Disease Activity Score of 28 Joints (DAS28), Female Self-Advocacy in Cancer Survivorship Scale, PedsQL 4.0, and Adolescent Mediation Barriers Scale Z. ^2^ Readability algorithms include: Flesch Reading Ease Formula, Flesch Kincaid Grade Level Formula, Gunning Fox Index, Coleman Liau Index, Simple Measure of Gobbledygook (SMOG), Automated Readability Index, and Linsear Write Formula. ^3^ Health literacy instruments include Health Literacy Questionnaire (HLQ), Health Literacy Assessment Tool for Identifying Facilitating Factors and Barriers to Information, Care, and Services (HLE2), and eHealth Literacy Scales (eHEALS).

## Data Availability

The search strategy (including search terms) is delineated in Materials and Methods (Section 2.2), Figure 1 depicts the PRISMA diagram delineating the article selection process, and Supplemental Table S1 provides the data extraction table for all included articles.

## Supplementary Materials

The following supporting information can be downloaded at https://www.mdpi.com/article/10.3390/healthcare11192615/s1, Table S1: Data extraction table summarizing included studies.
